# Comparing three induction chemotherapy regimens for patients with locoregionally advanced nasopharyngeal carcinoma based on TNM stage and plasma Epstein–Barr virus DNA level

**DOI:** 10.1186/s12885-020-6555-7

**Published:** 2020-02-03

**Authors:** Sai-Lan Liu, Xue-Song Sun, Hao-Jun Xie, Qiu-Yan Chen, Huan-Xin Lin, Hu Liang, Yu-Jing Liang, Xiao-Yun Li, Jin-Jie Yan, Chao Lin, Zhen-Chong Yang, Shan-Shan Guo, Li-Ting Liu, Qing-Nan Tang, Yu-Yun Du, Lin-Quan Tang, Ling Guo, Hai-Qiang Mai

**Affiliations:** 1 Collaborative Innovation Center for Cancer Medicine, Guangdong Key Laboratory of Nasopharyngeal Carcinoma Diagnosis and Therapy, Sun Yat-sen University Cancer Center; State Key Laboratory of Oncology in South China, 651 Dongfeng Road East, Guangzhou, 510060 People’s Republic of China; 2 Department of Nasopharyngeal Carcinoma, Sun Yat-sen University Cancer Center, 651 Dongfeng Road East, Guangzhou, 510060 People’s Republic of China; 3 Department of Radiation Oncology, Sun Yat-sen University Cancer Center, 651 Dongfeng Road East, Guangzhou, 510060 People’s Republic of China

**Keywords:** Nasopharyngeal carcinoma, Induction chemotherapy, Prognosis, Plasma Epstein–Barr virus

## Abstract

**Background:**

We compared the efficacy and toxicity of three IC regimens (TPF: taxanes, cisplatin, and 5-fluorouracil; TP: taxanes and cisplatin; and PF: cisplatin and 5-fluorouracil) followed by CCRT in locoregionally advanced NPC.

**Methods:**

The retrospective study involved 1354 patients with newly diagnosed stage III-IVA NPC treated with IC and CCRT. The median follow-up time in our cohort was 50 months. Based on EBV DNA level, all the patients with stage IV were divided into low- (pre-EBV DNA < 1500 copies) and high-risk group (pre-EBV DNA ≥ 1500 copies). Progression free survival (PFS), overall survival (OS), locoregional relapse free survival (LRFS), distant metastasis free survival (DMFS) and grade 3–4 toxicities were compared among different IC regimens. The survival rates were compared using log-rank test and a Cox proportional hazards model was used to perform multivariate analyses.

**Results:**

A multivariate analysis revealed TPF to be more effective than TP. Among stage III patients, no significant difference in clinical outcome between the different IC regimens was showed, while TPF was associated with significantly better survival conditions in the stage IV patients. A further subgroup analysis revealed that only patients with pre-EBV DNA ≥ 1500 copies could benefit from the application of TPF among stage IV NPC. In terms of acute toxicities, PF was associated with fewer grade 3/4 acute toxicities.

**Conclusions:**

In low-risk NPC patients, PF-based IC showed similar efficacy as TPF and TP but was associated with fewer grade 3/4 acute toxicities. In high-risk patients, however, the TPF regimen was superior to PF and TP, although grade 3/4 toxicities were more common with the TPF regimen.

## Background

Nasopharyngeal carcinoma (NPC) is a malignant disease arising from the nasopharyngeal epithelium. It is most endemic to Southern China, where 50–80 cases per 100,000 persons are reported each year [1]. Because of the radiosensitive nature of NPC and the typically deep-seated location of the lesions, radiation therapy (RT) is the primary treatment for NPC [2]. The development of modern RT has resulted in improved local control rates for NPC [3–5]. However, the prevention of distant metastasis in advanced NPC remains unsatisfactory and is the main cause of treatment failure [6]. Therefore, an effective treatment protocol is necessary to achieve better outcomes in these cases.

For non-metastatic locoregionally advanced NPC, concurrent chemoradiation therapy (CCRT) has been shown to be more effective than RT alone and has been accepted as the standard treatment for advanced NPC [7, 8]. Nevertheless, induction chemotherapy (IC) combined with the established CCRT regimen has recently attracted attention for the management of advanced NPC. The use of IC followed by definitive CCRT is associated with decreased distant metastases, which could improve clinical outcomes [9–15].

IC has been widely used in clinical practice; however, thus far, there is no consensus on the most suitable IC regimen. Therefore, it is important to evaluate the different IC regimens according to their efficacy and toxicity. Unfortunately, there was no large-scale clinical trial with convincing results to compare the efficacy of different IC regimen up to now [16]. To address this problem, in this study, we retrospectively analyzed 1354 NPC patients who received IC before concurrent chemotherapy. Taxanes, cisplatin, and 5-fluorouracil (TPF); cisplatin and 5-fluorouracil (PF); and taxanes and cisplatin (TP) were the most frequently used IC regimens in our center and were evaluated in this cohort. We especially analyzed the differences in patients’ survival outcomes in the three IC groups as well as the acute toxicity of the regimens. Besides, the plasma EBV DNA level has been proved to be useful in the prognostic prediction for NPC [17]. Accordingly, we divided patients in different risk level based on their pre-treatment EBV DNA and compared the curative effect of these three IC regimens in different subgroups, which was not reported in previous studies.

## Methods

### Patients

From 2008 to 2017, 1354 previously untreated NPC patients were enrolled in the study. The eligibility criteria for inclusion were newly diagnosed biopsy-proven NPC; receipt of first-line IC for at least 2 cycles followed by CCRT; Karnofsky performance score (KPS) > 70; adequate organ functions and with available hematological sample and EBV serology results. Key exclusion criteria were as the following: received palliative treatment; a history of malignancy; received previous anti-tumor treatment (radiotherapy, chemotherapy, or surgery [except diagnostic procedures]); the presence of lactation, pregnancy or severe coexisting illness.

The following examinations were performed for all patients: a complete physical examination, head and neck magnetic resonance imaging (MRI), chest radiography, abdominal sonography, electrocardiography, bone scan, nasopharyngoscopy, and complete blood count including differential cell counts, biochemical profile, and EBV serology. For partial patients, positron emission tomography/computed tomography (PET-CT) was also optionally performed to evaluate distant lesions. The study was approved by the Sun Yat-sen University Cancer Center Research Ethics Committee.

### Chemotherapy and RT

All patients received one of the following IC regimens: PF (comprising cisplatin [80–100 mg/m^2^, day 1] and 5-fluorouracil [800–1000 mg/m^2^, day 1–5, 120 h of continuous intravenous infusion]); TP (comprising docetaxel [75 mg/m^2^, day 1], paclitaxel [150–180 mg/m^2^, day 1] or paclitaxel liposome [150–180 mg/m^2^, day 1], and cisplatin [20–25 mg/m^2^/day, day 1–3]); and TPF (comprising docetaxel [60 mg/m^2^, day 1], paclitaxel [135 mg/m^2^, day 1] or paclitaxel liposome [135 mg/m^2^, day 1], cisplatin [20–25 mg/m^2^/day, days 1–3], and 5-fluorouracil [500–800 mg/m^2^, 120 h of continuous intravenous infusion]). All regimens were administered every 3 weeks over 2–4 cycles. RT was administered to the nasopharynx and neck by using intensity-modulated RT (IMRT) or two-dimensional RT (2D-CRT). IC was followed by concurrent cisplatin-based chemotherapy (80–100 mg/m^2^ every 3 weeks or 30–40 mg/m^2^ weekly) [7, 18]. Five daily fractions of a total dose of 68~70 Gy at about 2 Gy per fraction were prescribed per week. Other details of the IMRT plan were in line with the principles described in previous studies [19–21].

### Outcome and follow-up

The primary endpoint of our study was PFS, defined as the period from the first day of treatment to the date of disease progression or death from any cause. The other clinical endpoints were OS (defined as the period from the date of treatment to the date of death from any cause), LRFS (defined as the period from date of treatment to the date of local/regional relapse), and distant metastasis-free survival (DMFS), (defined as the time from date of treatment to the date of distant metastasis). Hematological reactions were evaluated for acute IC-associated toxicity, classified based on the National Cancer Institute Common Terminology Criteria for Adverse Events version 4.0, and compared between the groups. Physical examination, nasopharyngoscopy, and MRI of the head and neck were performed 3–6 months after RT completion. We evaluated tumor responses according to the Response Evaluation Criteria in Solid Tumors [22]. After treatment completion, the patients were evaluated every 3 months during the first 3 years and every 6 months thereafter until death. Nasopharyngoscopy, head and neck MRI, chest radiography, abdominal sonography, and plasma EBV DNA measurement were routinely performed.

### Statistical analysis

Statistical analyses were performed using SPSS package for Windows version 22.0 (Chicago, IL). Correlations between the different IC regimens and clinical characteristics of NPC were evaluated using the χ^2^ or Fisher’s exact test as appropriate. Kaplan–Meier survival curves were used to evaluate long-term survival; the survival rates were compared using log-rank test. A Cox proportional hazards model was used to perform multivariate analyses involving the following variables: age, sex, T stage, N stage, clinical stage, EBV DNA, and IC regimen. All analyses were two-sided. The level of significance was set at *P* < 0.05.

## Results

### Patient characteristics

New, consecutive patients (1354 patients including 335 [24.7%] females and 1019 [75.3%] males) diagnosed with non-metastatic NPC between June 2008 and November 2017 were included in this study. In the cohort, 1341 (99.0%) patients had WHO type III disease. The median patient age was 44 (8–74) years, and 772 (57.0%), 340 (25.1%), and 242 (17.9%) patients received TPF, PF, and TP chemotherapy, respectively. The median follow-up time was 27.3 months (range: 0.5–113.2 months) in the whole cohort, and 25.1 months (range: 3.1–90.8 months), 42.6 months (range: 3.2–113.2 months) and 25.7 months (range: 0.5–87.8 months) in TPF, PF and TP groups respectively. The cumulative cisplatin dose (CCD) was less than 200 mg/m^2^ in most patients (1059/1354, 78.2%). The patients’ other baseline characteristics are shown in Table 1.

**Table 1:** Patient demographics and clinical characteristics

		TPF(*n* = 772)	PF(*n* = 340)	TP(*n* = 242)	
Characteristics	No. (%)	No. (%)	No. (%)	No. (%)	*P value*
Age, years					0.307^a^
Median (range)	44(8–74)	43(8–74)	44(15–71)	46(18–71)	
< 45	698(51.6)	411(53.2)	171(50.3)	116(47.9)	
≥ 45	656(48.4)	361(46.8)	169(49.7)	126(52.1)	
Sex					0.580^a^
Female	335(24.7)	184(23.8)	91(26.8)	60(24.8)	
Male	1019(75.3)	588(76.2)	249(73.2)	182(75.2)	
Pathological type					0.217^b^
WHO type I	4(0.3)	3(0.4)	1(0.3)	0(0.0)	
WHO type II	9(0.7)	4(0.5)	5(1.5)	0(0.0)	
WHO type III	1341(99.0)	765(99.1)	334(98.2)	242(100)	
T stage^c^					0.199^a^
T1	18(1.3)	11(1.4)	5(1.5)	2(0.8)	
T2	149(11.0)	71(9.2)	49(14.4)	29(12.0)	
T3	665(49.1)	379(49.1)	168(49.4)	118(48.8)	
T4	522(38.6)	311(40.3)	118(34.7)	93(38.4)	
N stage^c^					< 0.001^a^
N0	35(2.6)	15(1.9)	11(3.2)	9(3.7)	
N1	315(23.3)	193(25.0)	57(16.8)	65(26.9)	
N2	695(51.3)	367(47.5)	197(57.9)	131(54.1)	
N3	309(22.8)	197(25.5)	75(22.1)	37(15.3)	
Clinical stage^c^					0.005^a^
III	612(45.2)	320(41.5)	167(49.1)	125(51.7)	
IVa-b	742(54.8)	452(58.5)	173(50.9)	117(48.3)	
EBV DNA					< 0.001^a^
≥ 1500	875(64.6)	514 (66.6)	231(67.9)	130(53.7)	
< 1500	479(35.4)	258 (33.4)	109(32.1)	112(46.3)	
RT technique					< 0.001^a^
2D RT	119(8.8)	7(0.9)	101(29.7)	11(4.5)	
IMRT	1235(91.2)	765(99.1)	239(70.3)	231(95.5)	
CCD (mg/m^2^)					0.127^a^
Median (range)	160(20–300)	160(25–300)	160(40–250)	160(20–300)	
≥ 200	295(21.8)	183(23.7)	63(18.5)	49(20.2)	
< 200	1059(78.2)	589(76.3)	277(81.5)	193(79.8)	

*Abbreviations*: *TPF* Taxanes plus cisplatin with fluorouracil, *PF* Cisplatin with fluorouracil, *TP* Taxanes with cisplatin, *EBV* Epstein–Barr virus, *CCD* Cumulative cisplatin dose during radiotherapy

^a^*P* values were calculated by the Chi-square test. ^b^*P* value calculated with Fisher’s exact test

^c^According to the 7th edition of UICC/AJCC staging system

### Survival analysis of patients treated with different IC regimens

**Fig. 1:**
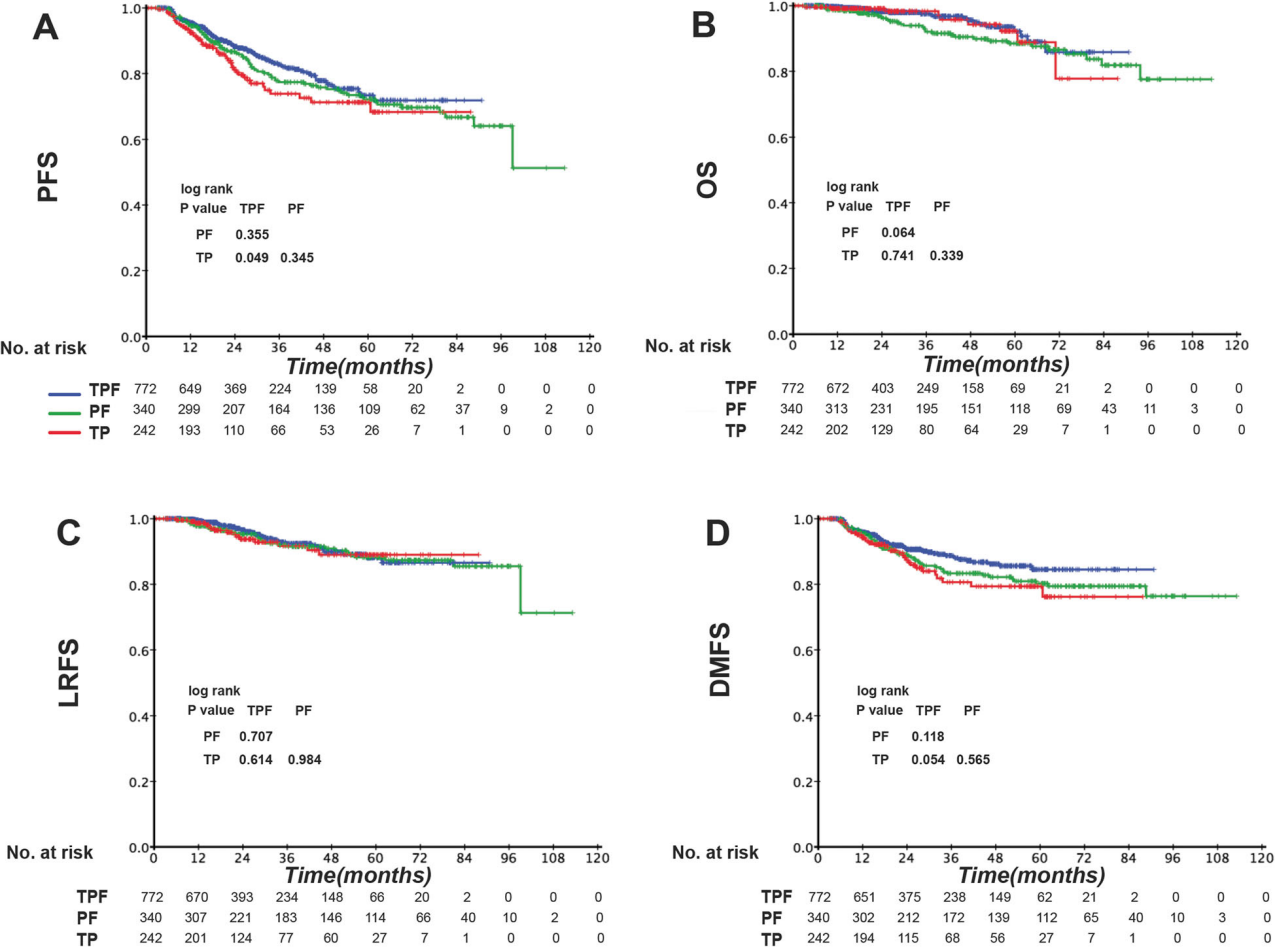
Kaplan–Meier (**a**) progression-free survival (PFS), **b** overall survival (OS), **c** locoregional relapse-free survival (LRFS), and **d** distant metastasis-free survival (DMFS) curves for the 1384 stage III-IVb NPC patients receiving induction TPF, PF, or TP

The 3-year PFS, OS, LRFS, and DMFS rates for the entire patient cohort were 79.4, 95.9, 88.0, and 85.6%, respectively. Regarding short-term tumor response, the complete response (CR)/partial response (PR) ratio was higher (79.7%) in TPF-receiving patients than in PF- and TP-receiving patients (67.2 and 71.4%, respectively; *P* < 0.001, Table 2). However, differences in long-term survival were only observed between TPF- and TP-treated patients and not between TPF- and PF-treated patients. Furthermore, the corresponding 3-year PFS, OS, LRFS and DMFS rates for TPF vs. PF vs. TP were 82.4% vs. 77.4% vs. 73.8% (P_TPF vs. PF_ = 0.335, P_TPF vs. TP_ = 0.049, P_PF vs. TP_ = 0.345; Fig. 1a), 97.2% vs. 92.1% vs. 97.0% (P_TPF vs. PF_ = 0.064, P_TPF vs. TP_ = 0.741, P_PF vs. TP_ = 0.339; Fig. 1b), 92.5% vs. 91.5% vs. 91.7% (P_TPF vs. PF_ = 0.707, P_TPF vs. TP_ = 0.614, P_PF vs. TP_ = 0.984; Fig. 1c), and 88.4% vs. 83.3% vs. 80.7% (P_TPF vs. PF_ = 0.118, P_TPF vs. TP_ = 0.054, P_PF vs. TP_ = 0.565; Fig. 1d) (Table 6 in Appendix 1). In the multivariate analysis, the following prognostic factors were evaluated: age, gender, pathological type, T stage, N stage, EBV DNA, and IC regimen. As shown in Table 3, TPF was associated with significantly better OS and DMFS than TP (OS: HR, 1.630; 95% CI, 0.151–2.308; *P* = 0.006; DMFS: HR, 1. 692; 95% CI, 1.115–2.569; *P* = 0.013), whereas not an independent prognostic factor compared with PF in all clinical outcome. As there was higher proportion of 2D-RT in PF group, we performed multivariate analysis involving RT method in PF group. As shown in the supplementary table, RT method was not an independent prognostic factor for all endpoints, indicating that its impact on survival conditions was relatively small (Table 7 in Appendix 2).

**Table 2:** Overall response rates at central review after the induction phase

	TPF(*n* = 707)	PF(*n* = 137)	TP(*n* = 147)	*P value*
Complete response	14(2.0%)	3(2.2%)	3(2.0%)	0.013
Partial response	548(77.5%)	89(65.0%)	102(69.4%)	
Stable disease	142(20.1%)	45(32.8%)	40(27.2%)	
Progressive disease	3(0.4%)	0 (0.0%)	2(1.4%)	

*P* value calculated with Fisher’s exact test

*Abbreviations*: *TPF* Taxanes plus cisplatin with fluorouracil, *PF* Cisplatin with fluorouracil, *TP* Taxanes with cisplatin

**Table 3:** Multivariable analysis of prognostic factors for III-IVb NPC patients

	Hazard ratio* (95% CI)	*P* value
Progression-free survival		
Age (y) (≥ 45 vs. < 45)	1.543(1.100–2.164)	0.012
Gender(F vs. M)	1.055(0.814–1.366)	0.686
T category (3–4 vs. 1–2)	1.280(0.846–1.937)	0. 242
N category (2–3 vs. 0–1)	1.357(0.977–1.886)	0.069
Overall stage (IVa-b vs. III)	1.487(1.133–1.951)	0.004
EBV DNA	1.579(1.151–2.164)	0.005
IC regimen; PF vs. TPF	1.189(0.880–1.605)	0.260
IC regimen; TP vs. TPF	1.630(1.151–2.308)	0.006
Overall survival		
Age (y) (≥ 45 vs. < 45)	0.892(0.510–1.560)	0.688
Gender(F vs. M)	1.846(1.115–3.055)	0.017
T category (3–4 vs. 1–2)	0.967(0.475–2.177)	0.927
N category (2–3 vs. 0–1)	1.161(0.620–2.177)	0.641
Overall stage (IVa-b vs. III)	1.606(0.950–2.715)	0.077
EBV DNA	3.881(1.657–9.090)	0.002
IC regimen; PF vs. TPF	1.604(0.917–2.804)	0.098
IC regimen; TP vs. TPF	1.571(0.719–3.436)	0.258
Locoregional relapse-free survival		
Age (y) (≥ 45 vs. < 45)	1.525(0.872–2.668)	0.139
Gender(F vs. M)	0.970(0.631–1.492)	0.891
T category (3–4 vs. 1–2)	1.189(0.598–2.364)	0.622
N category (2–3 vs. 0–1)	0.890(0.542–1.462)	0.645
Overall stage (IVa-b vs. III)	1.240(0.798–1.928)	0.339
EBV DNA	1.672(0.983–2.843)	0.058
IC regimen; PF vs. TPF	1.157(0.709–1.887)	0.559
IC regimen; TP vs. TPF	1.298(0.711–2.369)	0.395
Distant metastasis-free survival		
Age (y) (≥ 45 vs. < 45)	1.592(1.051–2.411)	0.028
Gender(F vs. M)	0.951(0.694–1.303)	0.756
T category (3–4 vs. 1–2)	1.428(0.852–2.393)	0.177
N category (2–3 vs. 0–1)	1.706(1.118–2.606)	0.013
Overall stage (IVa-b vs. III)	1.488(1.071–2.068)	0.018
EBV DNA	1.424(0.980–2.069)	0.063
IC regimen; PF vs. TPF	1.349(0.940–1.936)	0.104
IC regimen; TP vs. TPF	1.692(1.115–2.569)	0.013

A Cox proportional hazards regression model was used to detect variables individually without adjustment. All variables were transformed into categorical variables. HRs were calculated for age (years) (≥45 vs. < 45), sex (female vs. male), T stage (T3–4 vs. T1–2), N stage (N2–3 vs. N0–1), plasma EBV DNA before the first treatment (≥1500 copies/ml vs. < 1500 copies/ml), overall stage (IVa-b vs. III), and IC regimen (PF vs. TPF, TP vs. TPF)

*Abbreviations*: *CI* Confidence interval, *EBV* Epstein–Barr virus, *IC* Induction chemotherapy, *TPF* Taxanes plus cisplatin with fluorouracil, *PF* Cisplatin with fluorouracil, *TP* Taxanes with cisplatin

### Subgroup analysis according to the TNM stage and EBV level

**Fig. 2:**
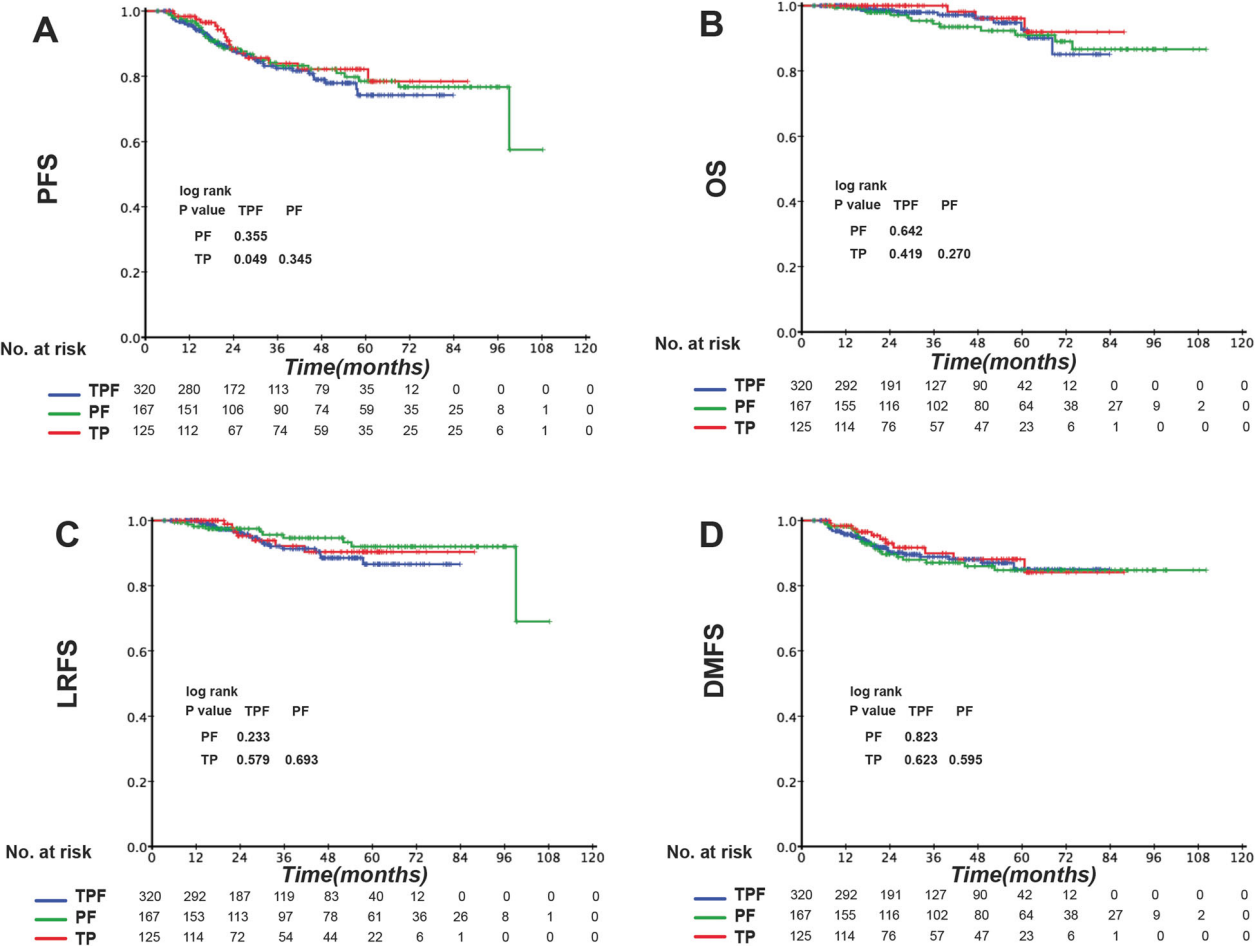
Kaplan–Meier (**a**) progression-free survival (PFS), **b** overall survival (OS), **c** locoregional relapse-free survival (LRFS), and **d** distant metastasis-free survival (DMFS) curves for the 612 stage III NPC patients receiving induction TPF, PF, or TP

**Fig. 3:**
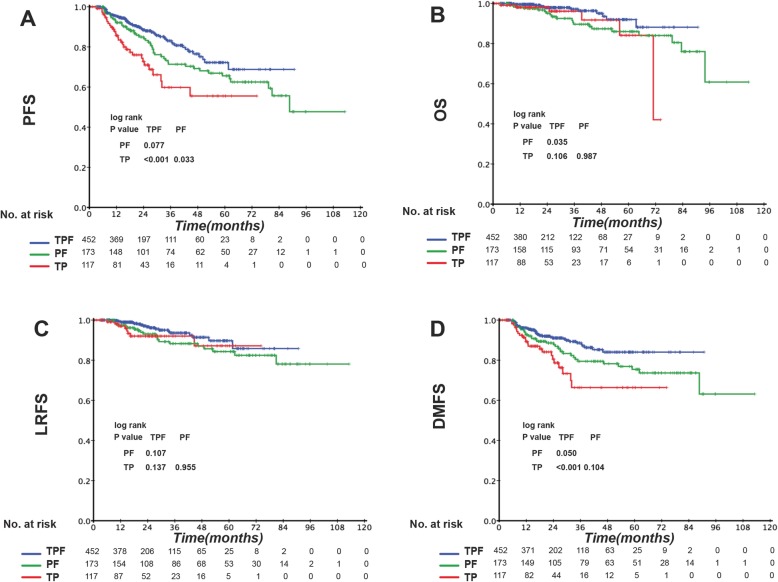
Kaplan–Meier (**a**) progression-free survival (PFS), (**b**) overall survival (OS), **c** locoregional relapse-free survival (LRFS), and **d** distant metastasis-free survival (DMFS) curves for the 742 stage IVa-b NPC patients receiving induction TPF, PF, or TP

**Fig. 4:**
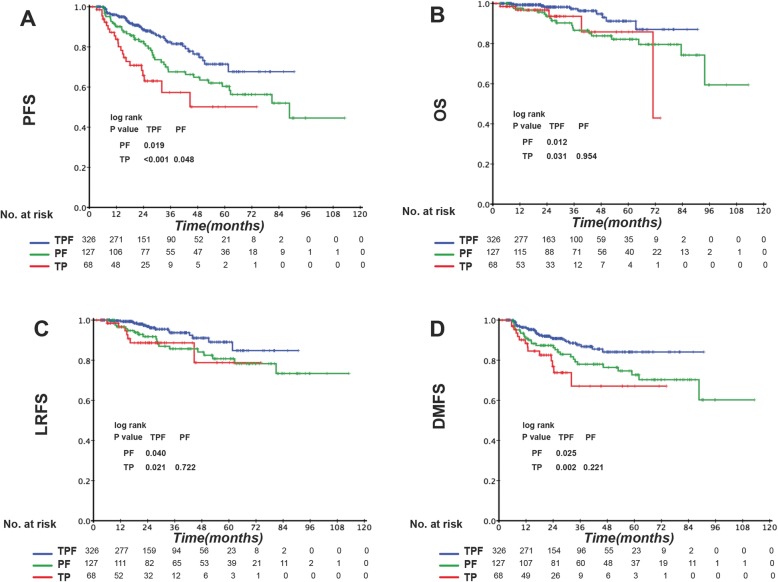
Kaplan–Meier (**a**) progression-free survival (PFS), (**b**) overall survival (OS), (**c**), locoregional relapse-free survival (LRFS), and (**d**) distant metastasis-free survival (DMFS) curves for the 521 stage IVa-b patients with EBV ≥ 1500 copies/ml NPC patients receiving induction TPF, PF, or TP

Patients at different TNM stages exhibited different tumor burdens and treatment failure rates. Thus, we divided the patients according to the TNM stage into stage III and IV disease groups (Table 8 in Appendix 3) and compared the prognostic impact of the IC regimens in the two groups. Among the three IC regimens, patients in TPF groups showed the highest complete response/ partial response rate after the induction phase (TPF vs. PF vs. TP: 79.5% vs. 67.2% vs. 71.4%, *P* = 0.013). Stage III patients showed no significant difference in clinical outcome between the different IC regimens (Fig. 2). However, in the IVA-IVB stage subgroup, TPF was associated with significantly better OS and DMFS than was PF and better PFS and DMFS than was TP (Fig. 3). EBV DNA is a prognostic factor for NPC patients. Therefore, we divided stage IV patients into low-risk and high-risk subgroups according to the EBV DNA level. Interestingly, prognostic factors differed between these two subgroups. Among low-risk patients (pre-EBV DNA < 1500 copies), the 3-year PFS, OS, LRFS, and DMFS rates in the different IC groups were similar and the survival curves were superimposable (data not shown). However, in the high-risk group (pre-EBV DNA ≥ 1500 copies), TPF was associated with significantly better PFS, OS, LRFS, and DMFS than were PF and TP. The 3-year PFS, OS, LRFS, and DMFS rates for TPF vs. PF vs. TP were 81.5% vs. 67.6% vs. 57.3% (P_TPF vs. PF_ = 0.019, P_TPF vs. T*P*_ < 0.001, P_PF vs. TP_ = 0.048 Fig. 4a), 97.3% vs. 86.6% vs. 85.8% (P_TPF vs. PF_ = 0.012, P_TPF vs. TP_ = 0.031, P_PF vs. TP_ = 0.954 Fig. 4b), 93.7% vs. 85.7% vs. 78.8% (P_TPF vs. PF_ = 0.040, P_TPF vs. T*P*_ = 0.021, P_PF vs. TP =_ 0.722 Fig. 4c), and 86.8% vs. 78.0% vs. 67.1% (P_TPF vs. PF_ = 0.025, P_TPF vs. TP_ = 0.002, P_PF vs. TP_ = 0.221 Fig. 4d) (Table 9 in Appendix 4).

As shown in Table 4, after adjusting for various factors, the IC regimen was established as an independent prognostic factor for PFS (PF vs. TPF: HR, 1.657; 95% CI, 1.079–2.544; *P* = 0.021; TP vs. TPF: HR, 3.222; 95% CI, 1.917–5.416: *P* < 0.001), OS (PF vs. TPF: HR, 2.608; 95% CI, 1.180–5.762; *P* = 0.018; TP vs. TPF: HR, 3.117; 95% CI, 1.051–9.244; *P* = 0.040), and DMFS (PF vs. TPF: HR, 2.978; 95% CI, 1.566–5.663; *P* = 0.001; TP vs. TPF: HR, 1.724; 95% CI, 1.076–2.763; *P* = 0.024). Clinical stage was also considered as a prognostic factor for DMFS.

**Table 4:** Multivariable analysis of prognostic factors for IVa-b patients with EBV DNA level ≥ 1500 copies/ml

	Hazard ratio* (95% CI)	*P* value
Progression-free survival		
Age (y) (≥ 45 vs. < 45)	1.171(0.802–1.710)	0.414
Gender(F vs. M)	1.421(0.857–2.357)	0.173
Clinical stage (IVb vs. IVa)	1.275(0.870–1.870)	0.213
IC regimen; PF vs. TPF	1.657(1.079–2.544)	0.021
IC regimen; TP vs. TPF	3.222(1.917–5.416)	< 0.001
Overall survival		
Age (y) (≥ 45 vs. < 45)	1.895(0.941–3.817)	0.074
Gender(F vs. M)	0.689(0.320–1.485)	0.342
Clinical stage (IVb vs. IVa)	0.814(0.401–1.651)	0.568
IC regimen; PF vs. TPF	2.608(1.180–5.762)	0.018
IC regimen; TP vs. TPF	3.117(1.051–9.244)	0.040
Locoregional relapse-free survival		
Age (y) (≥ 45 vs. < 45)	0.868(0.466–1.617)	0.594
Gender(F vs. M)	1.513(0.635–3.603)	0.350
Clinical stage (IVb vs. IVa)	0.919(0.490–1.724)	0.792
IC regimen; PF vs. TPF	2.091(1.043–4.191)	0.038
IC regimen; TP vs. TPF	2.626(0.490–1.724)	0.037
Distant metastasis-free survival		
Age (y) (≥ 45 vs. < 45)	1.103(0.692–1.756)	0.680
Gender(F vs. M)	1.210(0.675–2.171)	0.522
Clinical stage (IVb vs. IVa)	1.762(1.046–2.967)	0.033
IC regimen; PF vs. TPF	2.978(1.566–5.663)	0.001
IC regimen; TP vs. TPF	1.724(1.076–2.763)	0.024

*Abbreviations*: *CI* Confidence interval, *IC* Induction chemotherapy, *TPF* Taxanes plus cisplatin with fluorouracil, *PF* Cisplatin with fluorouracil, *TP* = Taxanes with cisplatin

A Cox proportional hazards regression model was used to detect variables individually without adjustment. All variables were transformed into categorical variables. HRs were calculated for age (years) (≥45 vs. < 45 years), sex (female vs. male), clinical stage (IVb vs. IVa), and IC regimen (PF vs. TPF, TP vs. TPF)

### Acute toxicity profile

In terms of acute toxicity during the IC period, patients in the TPF group experienced significantly more toxic effects than patients in the PF group, but similar toxic effects as patients in the TP group: leukocytopenia (grade 0–2: 75% vs. 95.3% vs. 82.2%; grade 3–4: 25.0% vs. 4.7% vs. 17.1%; *P* < 0.001) and neutropenia (grade 0–2: 57.4% vs. 87.1% vs. 64.5%; grade 3–4: 42.6% vs. 12.9% vs. 35.5%; *P* < 0.001). Intergroup differences in other acute toxicities such as anemia, ALT level increase, AST level increase, and BUN increase were not significant (Table 5).

**Table 5:** Grade 3–4 acute toxicities due to IC between the three arms

Adverse event (toxicity grade)	TPF(n = 772)		PF(n = 340)		TP(n = 242)		*P*
	0–2(%)	3–4(%)	0–2(%)	3–4(%)	0–2(%)	3–4(%)	
Leukocytopenia	579(75.0)	193(25.0)	324(95.3)	16(4.7)	199(82.2)	43(17.1)	< 0.001^a^
Neutropenia	443(57.4)	329(42.6)	296(87.1)	44(12.9)	156(64.5)	86(35.5)	< 0.001^a^
Anemia	763(98.8)	9(1.2)	339(99.7)	1(0.3)	240(99.2)	2(0.8)	0.441 ^b^
Thrombocytopenia	765(99.1)	7(0.9)	338(99.4)	2(0.6)	239(98.8)	3(1.2)	0.672^b^
ALT increase	763(99.0)	8(1.0)	337(99.1)	3(0.9)	239(98.8)	3(1.2)	0.871 ^b^
AST increase	771(99.9)	1(0.1)	339(99.7)	1(0.3)	241(99.6)	1(0.4)	0.395^b^
Creatinine increase	771(99.9)	1(0.1)	339(99.7)	1(0.3)	242(100)	0(0.0)	0.675^b^
BUN increase	771(99.9)	1(0.1)	340(100)	0(0.0)	240(99.2)	2(0.8)	0.134^b^

*Abbreviations*: *IC* Induction chemotherapy, *TPF* Taxanes plus cisplatin with fluorouracil, *PF* Cisplatin with fluorouracil, *TP* Taxanes with cisplatin, *ALT* Alanine aminotransferase, *AST* Aspartate aminotransferase, *BUN* blood urea nitrogen

^a^*P* values were calculated by Chi-square test. ^b^*P* value calculated with Fisher’s exact test

## Discussion

Distant metastasis remains a critical issue in cases of advanced NPC [23, 24], and IC could facilitate the eradication of micro-metastatic lesions and reduce locoregional failure. With the increasing evidence for the effectiveness of IC followed by CCRT for advanced NPC [10, 25–28], IC is being widely used in clinical settings. TPF, PF, and TP are the three induction regimens most frequently used for advanced NPC worldwide, and all of them can improve survival in patients with locoregionally advanced NPC [10, 25, 26]. Our study indicates that TPF is the best choice among these three induction regimens for lowering the distant metastasis rate and improving the overall survival (OS) rate in high-risk NPC patients (IVa-b NPC patients with EBV DNA ≥ 1500 copies/ml).

Our data showed that most patients in the PF and TP groups were treated in the early years while the recent ones were distributed to the TPF group. The TPF IC regimen is commonly used in advanced head and neck cancer [29, 30]. In comparison with the standard PF regimen, regimens including taxanes, which are microtubule-stabilizing drugs that have been extensively used as effective chemotherapeutic agents for solid tumor treatment [31], showed significantly better PFS and OS and higher CR rates in head and neck cancers [29, 30, 32]. In another study, TPF demonstrated long-term survival benefits over PF in locally advanced head and neck cancer [33]. Long-term follow-up data confirm that TPF could increase larynx preservation and larynx dysfunction-free survival [34]. Undoubtedly, these benefits may also apply to NPC. Compared with the TP regimen, regimens including fluorouracil may also provide therapeutic gains. Lee et al. [35] found that the fluorouracil dose during the adjuvant phase was associated with significantly improved distant failure-free survival in a combined analysis of NPC-9901 and NPC-9902. This effect may also be present in the induction phase. Therefore, a combination of these three active agents seems to be the most effective regimen to provide optimal therapeutic benefit. Based on the above reasons, more clinicians preferred to select the TPF regimen recently.

Previous studies have suggested that TPF is superior to TP and PF for NPC patients. One report [16] demonstrated that the TP regimen may be sufficient for patients receiving a CCD ≥ 200 mg/m^2^, while TPF may be superior to TP and PF for patients receiving a CCD < 200 mg/m^2^. In Liu’s study [36], the TPF regimen yielded better long-term survival for patients with locoregionally advanced NPC in comparison with the PF regimen. In another study [37], TPF showed an improved early response of lymph node size reduction in comparison with the PF and TP regimens. These findings support the results of the present study, in which IC with TPF showed the best short-term tumor response and provided survival benefits compared with those of TP in all cases of locally advanced nasopharyngeal carcinoma.

However, one of the major limitations of previous studies was that they did not present data for plasma EBV DNA levels, which is an important prognostic factor for NPC patients and, in combination with the TNM stage, could identify patients with locoregionally advanced NPC who are at a high risk of locoregional recurrence and distant metastasis [17]. In a subgroup analysis stratified by clinical stage and EBV DNA levels, we observed an interesting scenario. Among patients with stage III NPC, survival outcomes were comparable between the three groups. However, among patients with stage IVa-b NPC, TPF could not only reduce distant metastasis but also prolong PFS and OS in comparison with TP and PF. Thus, TPF could reduce distant metastasis and improve the local control rate for patients (IVa-b) with a high tumor burden in comparison with TP and PF. A subgroup analysis of stage IVa-b NPC patients stratified by EBV DNA levels showed no survival benefit of TPF over PF and TP among low-risk patients (IVa-b with EBV DNA < 1500 copies/ml). However, in high-risk patients (IVa-b with EBV DNA ≥ 1500 copies/ml), TPF achieved the best outcomes among the three induction regimens for improving the survival rate and lowering the distant metastasis rate. Hence, the more effective regimen, TPF, is particularly important for high-risk (IVa-b with EBV DNA ≥ 1500 copies/ml) patients.

Obviously, a combination of three agents produced more grade 3–4 toxicities. Notably, leukocytopenia and neutropenia were significantly higher in the TPF arm (25.0 and 42.6%, respectively) and TP arm (17.1 and 35.5%, respectively) than in the PF arm (4.7 and 12.9%, respectively), whereas other toxicities were common in the three arms. This difference should be attributable to taxanes since the most common adverse event after taxane therapy is myelosuppression. Finally, the results showed that survival outcomes were comparable between the three groups in low-risk NPC patients (stage III and IVa-b with EBV DNA < 1500 copies/ml), and the incidences of leukocytopenia and neutropenia were lower in the PF arm than in the TPF and TP arms. These findings indicate that PF-based IC has similar efficacy to TPF and TP in low-risk NPC patients (stage III and IVa-b with EBV DNA < 1500 copies/ml) but is associated with fewer grade 3/4 acute toxicities.

The data reported in this article also have several limitations. First, there was an inevitable bias caused by the retrospective nature of this study. Because of the selective bias, there were certain clinicopathologic differences among the patients receiving different IC regimens. Besides, the follow-up periods were also various in different IC groups. Although all potential prognostic factors were included in the multivariate analyses to avoid confounding effects, the credibility of our conclusions was still affected to some extent. Second, although our cohort is likely to be representative of the majority of patients diagnosed with NPC in South China, this was a single-center study. A multi-center study is needed to fully compare different IC regimens for locoregionally advanced nasopharyngeal carcinoma.

## Conclusions

In summary, our study concluded that an induction TPF regimen was superior to TP and PF regimens for high-risk (IVa-b with EBV DNA ≥ 1500 copies/ml) NPC, although grade 3–4 toxic events were more common but tolerable in the TPF arm. However, PF-based IC has similar efficacy to TPF and TP in low-risk NPC patients (stage III and IVa-b with EBV DNA < 1500 copies/ml) but is associated with fewer grade 3/4 acute toxicities. Further studies are needed to validate our findings.

## Data Availability

The datasets used and/or analysed during the current study are available from the corresponding author on reasonable request.

## Appendix 1

**Table 6:** Three-year PFS, OS, LRFS, DMFS based on different IC regimens in III-IVb patients

	TPF (%) *n* = 772	PF (%) *n* = 340	TP (%) *n* = 242	*P* value
Progression-free survival				
Failures	108(14.0%)	42(22.6%)	47 (19.4%)	0.127
Rate at 3 years	82.4% (78.9–85.9)	77.4% (72.3–82.5)	73.8% (66.7–80.9)	
Overall survival				
Deaths	23(3.0%)	32 (9.4%)	9(3.7%)	0.029
Rate at 3 years	97.2% (95.6–98.8)	92.1% (88.8–95.4)	97.0% (93.9–100)	
Locoregional relapse-free survival				
Locoregional failure	40(5.2%)	30 (8.8%)	15 (6.2%)	0.835
Rate at 3 years	92.5% (90.0–95.0)	91.5% (88.0–95.0)	91.7% (87.2–96.2)	
Distant metastasis-free survival				
Distant failures	72(9.3%)	53 (15.6%)	33 (13.6%)	0.103
Rate at 3 years	88.4% (85.7–91.1)	83.3% (78.8–87.8)	80.7% (74.0–87.4)	

Data are *n* (%) or rate (95% CI). *P* values were calculated with the unadjusted log-rank test

## Appendix 2

**Table 7:** Multivariable analysis of prognostic factors for III-IVb NPC patients treated with PF regimen

	HR (95%CI)	*P*-value value
Progression-free survival		
Age (y) (≥ 45 vs. < 45)	1.319(0.839–2.074)	0.231
Gender (F vs. M)	1.496(0.844–2.651)	0.168
T category (3–4 vs. 1–2)	1.556(0.755–3.208)	0.231
N category (2–3 vs. 0–1)	1.736(0.897–3.361)	0.101
Overall stage (IVa-b vs. III)	1.711(1.047–2.796)	0.032
EBV DNA (< 1500 vs. ≥1500)	2.327(1.223–4.427)	0.010
RT method (IMRT vs. 2D-RT)	1.159(0.910–1.477)	0.232
Overall survival		
Age (y) (≥ 45 vs. < 45)	2.067(0.986–4.33)	0.054
Gender (F vs. M)	0.710(0.329–1.535)	0.384
T category (3–4 vs. 1–2)	0.921(0.335–2.533)	0.874
N category (2–3 vs. 0–1)	1.150(0.446–2.966)	0.772
Overall stage (IVa-b vs. III)	1.998(0.911–4.384)	0.084
EBV DNA (< 1500 vs. ≥1500)	5.091(1.209–21.430)	0.027
RT method (IMRT vs. 2D-RT)	0.875(0.660–1.290)	0.244
Locoregional relapse-free survival		
Age (y) (≥ 45 vs. < 45)	1.035(0.500–2.140)	0.927
Gender (F vs. M)	0.866(0.378–1.986)	0.734
T category (3–4 vs. 1–2)	1.136(0.371–3.479)	0.823
N category (2–3 vs. 0–1)	0.812(0.334–1.975)	0.646
Overall stage (IVa-b vs. III)	2.002(0.890–4.502)	0.093
EBV DNA (< 1500 vs. ≥1500)	3.431(1.032–11.400)	0.044
RT method (IMRT vs. 2D-RT)	1.213(0.818–1.801)	0.337
Distant metastasis-free survival		
Age (y) (≥ 45 vs. < 45)	0.913(0.531–1.571)	0.742
Gender (F vs. M)	2.125(0.993–4.548)	0.052
T category (3–4 vs. 1–2)	2.041(0.791–5.265)	0.140
N category (2–3 vs. 0–1)	2.353(0.984–5.626)	0.054
Overall stage (IVa-b vs. III)	1.619(0.902–2.905)	0.107
EBV DNA (< 1500 vs. ≥1500)	1.912(0.927–3.946)	0.079
RT method (IMRT vs. 2D-RT)	1.242(0.919–1.679)	0.158

A Cox proportional hazards regression model was used to detect variables individually without adjustment. All variables were transformed into categorical variables. HRs were calculated for age (years) (≥45 vs. < 45), sex (female vs. male), T stage (T3–4 vs. T1–2), N stage (N2–3 vs. N0–1), plasma EBV DNA before the first treatment (≥1500 copies/ml vs. < 1500 copies/ml), overall stage (IVa-b vs. III), and RT method (IMRT vs. 2D-RT)

*Abbreviations*: *CI* Confidence interval, *EBV* Epstein–Barr virus, *PF* Cisplatin with fluorouracil

## Appendix 3

**Table 8:** Patient demographics and clinical characteristics

		III				IVA-IVB		
Characteristic	TPF(*n* = 320) No. (%)	PF(*n* = 167) No. (%)	TP(*n* = 125) No. (%)	*P*	TPF(*n* = 452) No. (%)	PF(*n* = 173) No. (%)	TP(*n* = 117) No. (%)	*P*
Age, y				0.106^a^				0.865^a^
Median (range)	42(13–70)	44(18–68)	46 (19–64)		45(8–74)	45(15–71)	46 (18–71)	
< 45	186(58.1)	89(53.3)	59(47.2)		225(49.8)	82(47.4)	57(48.7)	
≥ 45	134(41.9)	78(46.7)	66(52.8)		227(50.2)	91(52.6)	60(51.3)	
Gender				0.151^a^				0.668^a^
Female	78(24.4)	53(31.7)	29(26.1)		106 (23.5)	38(22.0)	31(26.5)	
Male	242(75.6)	114(68.3)	96(76.8)		346(76.5)	135(78.0)	86(73.5)	
Pathological type			0.660^b^				0.370^b^	
WHO type I	0(0)	0(0)	0(0)		3(0.7)	1(0.6)	0(0)	
WHO type II	2(0.6)	2(1.2)	0(0)		2(0.4)	3(1.7)	0(0)	
WHO type III	318(99.4)	165(98.8)	125(100)		447(98.9)	169(97.7)	117(100)	
T stage^c^				0.049^b^				0.426^a^
T1	4(1.3)	3(1.8)	1(0.8)		7(1.5)	2(1.2)	1(0.9)	
T2	41(12.8)	38(22.8)	23(18.4)		30(6.6)	11(6.4)	6(5.1)	
T3	275(85.9)	126(75.4)	101(80.8)		104(48.1)	168(48.4)	118(47.6)	
T4	0 (0)	0 (0)	0 (0)		311(39.5)	118(34.0)	93(37.5)	
N stage^c^				< 0.001^a^				0.047 ^a^
N0	6(1.9)	1(0.6)	5(4.0)		9(2.0)	10(5.8)	4(3.4)	
N1	95(29.7)	17(10.2)	32(25.6)		98(21.7)	40(23.1)	33(28.2)	
N2	219(68.4)	149(89.2)	88(70.4)		148(432.7)	48(27.7)	43(36.8)	
N3	0 (0)	0 (0)	0 (0)		197(43.6)	75(43.4)	37(31.6)	
EBV DNA				0.085^a^				0.007 ^a^
≥ 1500	188(58.8)	104(62.3)	62(49.6)		326(72.1)	127(73.4)	68(58.1)	
< 1500	132 (41.3)	63(37.7)	63(50.4)		126(27.9)	46(26.6)	49(41.9)	
RT technique			< 0.001^a^				< 0.001 ^b^	
2D RT	5(1.6)	52(31.1)	9(7.2)		2(0.4)	49(28.3)	2(1.7)	
IMRT	315(98.4)	115(68.9)	116(92.8)		450(99.6)	124(71.7)	115(98.3)	
CCD (mg/m2)				0.032 ^a^				0.791 ^a^
Median (range)	160(68–300)	160(40–250)	160(60–300)		160(25–300)	160(50–240)	160(20–300)	
≥ 200	85(26.6)	27(16.3)	27(21.6)		98(21.7)	36(20.8)	22(18.8)	
< 200	235(73.4)	140(83.8)	98(78.4)		354(78.3)	137(79.2)	95(81.2)	

*Abbreviations*: *TPF* Taxanes plus cisplatin with fluorouracil, *PF* Cisplatin with fluorouracil, *TP* Taxanes with cisplatin, *EBV* Epstein–Barr virus, *CCD* Cumulative cisplatin dose during radiotherapy

^a^*P* values were calculated by the Chi-square test. ^b^*P* value calculated with Fisher’s exact test

^c^According to the 7th edition of the UICC/AJCC staging system

## Appendix 4

**Table 9:** Three-year PFS, OS, DMFS, LRFS based on different IC regimens in IVa-b patients of EBV ≥ 1500 copies/ml

	TPF (%) *n* = 326	PF (%) *n* = 127	TP (%) *n* = 68	*P* value
Progression-free survival				
Failures	46(14.1%)	42(33.0%)	22 (32.4%)	< 0.001
Rate at 3 years	81.5% (75.6–87.4)	67.6% (58.2–77.0)	57.3%(41.0–73.6)	
Overall survival				
Deaths	10(3.1%)	19 (15.0%)	5(7.3%)	0.029
Rate at 3 years	97.3% (94.8–99.8)	86.6%(79.7–93.5)	85.8%(69.7–102)	
Locoregional relapse-free survival				
Locoregional failure	15(4.6%)	19 (15.0%)	7 (10.3%)	0.032
Rate at 3 years	93.7% (90.0–97.4)	85.7%(78.6–92.8)	78.8%(59.0–98.6)	
Distant metastasis-free survival				
Distant failures	31(9.5%)	28 (22.0%)	14 (20.6%)	0.003
Rate at 3 years	86.8% (81.9–91.7)	78.0%(69.8–86.2)	67.1%(49.9–84.3)	

Data are *n* (%) or rate (95% CI). *P* values were calculated with the unadjusted log-rank test

## Abbreviations

2D-CRT: Two-dimensional radiotherapy
CCD: Cumulative cisplatin dose
CCRT: Concurrent chemoradiotherapy
CIs: Confidence intervals
DMFS: Distant metastasis-free survival
EBV: Epstein–Barr Virus
HRs: Hazard ratios
IC: Induction chemotherapy
IMRT: Intensity-modulated radiotherapy
KPS: Karnofsky performance score
LRFS: Locoregional relapse-free survival
MRI: Magnetic resonance imaging
NPC: Nasopharyngeal carcinoma
OS: Overall survival
PET/CT: Positron emission tomography/computed tomography
PF: Cisplatin and 5-fuorouracil)
PFS: Progression-free survival
RT: Radiotherapy
TP: Taxanes and cisplatin
TPF: Taxanes, cisplatin and 5-fuorouracil
WHO: World Health Organization
